# A novel lncRNA‐mediated *trans*‐regulatory mechanism in the development of cleft palate in mouse

**DOI:** 10.1002/mgg3.522

**Published:** 2018-12-12

**Authors:** Xuan Shu, Shenyou Shu, Hongqiu Cheng

**Affiliations:** ^1^ The Cleft Lip and Palate Treatment Center Second Affiliated Hospital of Shantou University Medical College Shantou China; ^2^ Department of Infectious Diseases Second Affiliated Hospital of Shantou University Medical College Shantou China

**Keywords:** cleft palate, EMT, lncRNA, TF, *trans*‐regulatory network

## Abstract

**Background:**

Increasing evidence indicates that long non‐coding RNAs (lncRNAs) play crucial regulatory roles in epithelial–mesenchymal transition (EMT). However, the regulatory mechanisms during EMT of the medial edge epithelium (MEE) remain elusive. The aim of this work is to reveal a novel lncRNA‐regulated dysfunction of EMT involved in the development of cleft palate (CP).

**Methods:**

C57BL/6 J mice at embryonic gestation day 14.5 (*n* = 6, 3 case samples vs. 3 control samples) were used to establish the CP model for lncRNA–mRNA co‐expression profile analysis after high‐throughput sequencing. Functional predictions for the differentially expressed lncRNA–mRNA co‐expression with transcription factor (TF)‐target gene relationship Gene Ontology/Kyoto Encyclopedia of Genes and Genomes pathway (GO/KEGG) analyses identified the regulatory “lncRNA–TF‐target gene” *trans *model.

**Results:**

A total of 583 differentially expressed lncRNAs and 703 differentially expressed mRNAs were identified. The results of *trans *analysis revealed that some TFs (*LEF1*, *SMAD4,* and *FOXD3*) regulate lncRNAs and gene expression. Finally, we identified the *NONMMUT034790.2‐LEF1‐SMAD7* co‐expression *trans*‐regulatory network that might be associated with CP.

**Conclusions:**

Our results revealed that *NONMMUT034790.2 *might be a novel epigenetic biomarker in CP. The integration of lncRNA modulators into *trans*‐regulatory networks will further enhance our understanding of lncRNA functions and regulatory mechanisms during palatal fusion in ATRA‐induced mouse CP.

## 1 INTRODUCTION

Cleft palate (CP) is a common congenital birth defect in the oral and craniofacial region that results in feeding, speech, and hearing difficulties and occurs in approximately 1.7 in 1,000 live births worldwide (Mossey, et al., 2009). It may originate from disruptions in epithelial–mesenchymal transition (EMT) of the medial edge epithelium (MEE) during palate shelf fusion, including an imbalance in MEE apoptosis, post‐fusion rupture, or failure of mesenchyme consolidation (Choi, et al. 2011). The palatal shelves grow into the midline and palatal fusion occurs at embryonic gestation day 14.5 (E14.5) in mice. Any imbalance in embryonic palatal mesenchyme cell proliferation and apoptosis can result in CP formation (Nawshad, 2010; Rice, 2005; Thiery & Sleeman, 2006). A previous study showed that *LEF1 *(OMIM 153245) and *SMAD7 *(OMIM 602932) were involved in CP and palate formation (Mitra, et al., 2016; Nawshad, 2010; Rice, 2005; Thiery & Sleeman, 2006), but the underlying palatogenesis and potential regulatory mechanisms are still unclear.

Long non‐coding RNAs (lncRNAs) are defined as transcripts longer than 200 bp with no apparent protein‐coding potential (Chakraborty, et al. 2014; Li, Eichten, et al., 2014). lncRNAs play important roles in regulating gene expression at epigenetic, transcriptional, and post‐transcriptional levels during cell proliferation and differentiation, cell growth, and apoptosis (Zhu, Fu, Wu, & Zheng, 2013). Moreover, increasing evidence suggests that altered expression of lncRNAs could be associated with the genesis and progression of many diseases and conditions including CP (Geng, et al., 2013; Orom, et al., 2010; Wapinski & Chang, 2011). However, the specific role of lncRNAs in palatogenesis and potential regulatory mechanism during EMT of the MEE during palate shelf fusion involved in CP has not been reported.

Therefore, in the present study, we hypothesized that lncRNAs might be differentially expressed and might act in a direct or indirect manner during palate shelf fusion. To address this hypothesis, we first established a CP model using C57BL/6 J mice after treatment with all‐trans retinoic acid (ATRA) as reported previously (Qin, et al., 2014; Shu, et al. 2018). Then, lncRNA–mRNA co‐expression analysis was performed in E14.5 palatal tissues to assess the “lncRNA‐transcription factor (TF)‐target gene” by *trans*‐regulatory mechanisms. ATRA is a metabolite of vitamin A and mediates normal pattern formation during embryogenesis (Ackermans, et al. 2011). Abnormally high concentrations of ATRA were reported to induce fetal malformations, including CP, in both experimental animals and humans (Cuervo, et al., 2002). Furthermore, GO/KEGG analyses were performed for functional annotations of the differential expressions. lncRNAs and mRNAs identified the *trans‐*regulatory network of *NONMMUT034790.2 ‐* *LEF1 *(OMIM 153245) *‐* *SMAD7 *(OMIM 602932)*.* Finally, qPCR was used to verify the *NONMMUT034790.2‐ *(OMIM 153245) *‐* *SMAD7 *(OMIM 602932) expression level. The results of this study provide novel insights into the molecular mechanisms underlying mouse palate development and malformation, as in CP.

## 2 METHODS

### 2.1 Ethics, animals, and treatment

C57BL/6 J mice, weighing 20–28 g and 8–10 weeks of age, were purchased from Beijing Vital River Laboratory Animal Technology Co. Ltd. (Beijing, China). In this study, pregnant mice were administered ATRA (Sigma‐Aldrich. St. Louis., MO, USA) (70 mg/kg) by gavage at embryonic gestation day 10.5 (E10.5) to establish a cleft palate (CP) model in C57BL/6 J mice. Control groups were given an equivalent volume of corn oil. An ATRA‐induced mouse cleft palate model was established (*n* = 6, 3 case samples vs. 3 control samples), and palatal shelf tissues were collected and stored as our research group reported previously at E14.5 (Qin, et al., 2014; Shu, et al. 2018). The animal study protocol was approved by the Laboratory Animal Ethical Committee of Medical College of Shantou University (SUMC2015‐106, Shantou, China), and the experiments were carried out in accordance with the animal care guidelines of the US National Institutes of Health.

### 2.2 RNA extraction, library preparation, and RNA‐seq

Total RNA was extracted using a mirVana^TM^ miRNA isolation kit (Ambion, Invitrogen, Carlsbad, CA) following the manufacturer's protocol. RNA integrity was evaluated using Agilent 2100 Bioanalyzer (Agilent Technologies, Santa Clara, CA, USA). Samples with an RNA integrity number (RIN) ≥7 were subjected to subsequent analysis. Libraries were constructed using TruSeq Stranded Total RNA with Ribo‐Zero Gold (Illumina Inc., San Diego, CA) according to the manufacturer's instructions. The libraries were then sequenced on the Illumina sequencing platform by the Shanghai Oebiotech Co. Ltd (OE2016H1266YE, Shanghai, China) (HiSeqTM 2500), and 125/150 bp paired‐end reads were generated.

### 2.3 Sequence data processing

The raw data file obtained by high‐throughput sequencing was stored in FASTQ (fq) file format. After adaptor sequences and low‐quality sequences were removed from the original reads, the high‐quality clean reads were mapped to the Mouse Genome (ftp://ftp.ensembl.org/pub/release-84/fasta/mus_musculus/dna/Mus_musculus. GRCm38.dna.toplevel.fa.gz) with Tophat2 (Kim, et al., 2013; Trapnell, et al., 2010) for mRNA identification. Constructed transcripts were compared with Ensembl mouse gene annotation (ftp://ftp.ensembl.org/pub/release-84/gff3/mus_musculus/Mus_musculus.GRCm38.84.chr.gff3.gz) to identify expressed mRNAs using Cuffcompare (Trapnell, et al., 2012). Based on the alignment files and mapped reads results of each sample, the transcript was reconstructed using a probabilistic model with the Cufflinks package (Trapnell, et al., 2012). The reconstructed transcript of each sample was sorted and merged with Cuffmerge to generate a transcript collection that represented the transcript of samples in this study. Retained transcripts were then assessed for their coding potential and transcripts that possessed an open reading frame with the ability to code for a peptide of 100 or more amino acids were identified using CPC (Kong, et al., 2007), CNCI (Sun, et al., 2013), Pfam (Finn, et al., 2014), and PLEK (Li, Zhang, & Zhou, 2014) (*p* < 0.05, fold enrichment >2, and log_2_FC >1).

### 2.4 Differential mRNA and lncRNA expression analyses

To compare the expression level of a gene across samples, read counts obtained from the RNA‐seq data were normalized as fragments per kilobase of transcript per million mapped fragments (FPKM) (Mortazavi, Williams, McCue, Schaeffer, & Wold, 2008) with Bowtie 2 (Langmead and Steven, 2012) and eXpress (Roberts & Pachter, 2013) software packages. FPKM was used to identify differentially expressed genes in case and control, and then, the FPKM in each sample was compared. The differences in gene expression (mRNA or lncRNA) with a *p* < 0.05 and log_2_FC>1 were considered to significantly differentially expressed.

### 2.5 GO and KEGG analyses

After identifying the differentially expressed mRNAs and lncRNAs, we performed GO (Gene Ontology 2004) (http://geneontology.org/) and KEGG (Kanehisa, et al., 2006; Kanehisa, Goto, Kawashima, Okuno, & Hattori, 2004) (http://www.genome.jp/kegg/) analyses to assess and predict their functions (*p* < 0.05, fold enrichment >2).

### 2.6 lncRNA–mRNA co‐expression analyses

For each lncRNA, we calculated the Pearson correlation of its expression value with the expression value of each mRNA. For function prediction of lncRNAs, we calculated co‐expressed mRNAs for each differentially expressed lncRNA (Guttman, et al., 2009), and then performed GO/KEGG analyses for co‐expressed mRNAs. The enriched functional terms were used as the predicted functional term of a given lncRNA. The correlation coefficient between lncRNAs and mRNAs smaller than 0.05 and the absolute value of correlation greater than 0.7 were considered to have potential relevance.

### 2.7 “TF–lncRNA” network analyses

lncRNA sequences were mapped to the genome in the NONCODE(v5) database (Fang, et al., 2002). Jemboss software was used to examine the alignment of lncRNA and putative TF binding sequences (Carver and Mullan 2005) (http://emboss.sourceforge.net/Jemboss/). The genome browser database was used to build the network describing the relationships between TFs and lncRNAs (Casper, et al., 2018) (https://genome.ucsc.edu). Pearson correlation coefficient was used to authenticate the co‐expressed TF of lncRNAs (*p* < 0.05). Hypergeometric distribution test was used to calculate the GO/KEGG terms in the annotation of co‐expressed TF. The relationship of TF and lncRNA was generated using Cytoscape software (Kohl, et al. 2011) (*p* < 0.05, fold enrichment >2).

### 2.8 “lncRNA–TF‐target gene” network analyses

Based on the interactions of lncRNA and target genes assemble of TF/chromatin regulatory complex, the “lncRNA–TF‐target gene” network was constructed. The enrichment degree of the intersection was calculated by hypergeometric distribution. The TFs that were significantly related to the lncRNA were obtained, thus identifying the TF/chromatin regulatory factor that might be associated with lncRNAs. By means of hypergeometric distribution, each lncRNA can form multiple lncRNA–TF pairs. Each “lncRNA–TF” pair is the result of multiple gene enrichment. Based on *p*‐value distribution (low to high), the two elements relation graph used the regulatory relationship of the first 100 hits, and the three relation networks graph took the mapping relationship between the 10 hits. The “lncRNA–TF‐target gene” *trans*‐regulatory network was generated using Cytoscape software based on the “TF–lncRNA” network (Kohl, et al. 2011) (*p* < 0.05, fold enrichment >2).

### 2.9 qPCR validation

To validate the RNA‐seq data, qPCR was conducted in six individual samples. All reactions were carried out in triplicate for technical and biological repetitions. The qPCR primers used in this study are listed in Table 1. The mRNAs and lncRNAs relative expression levels were analyzed as described in a previous study, and the 2^−ΔΔCt^ method (Livak and Schmittgen 2001) was used to calculate the level of gene expression relative to the expression of *β*‐actin, as an internal control (*p* < 0.05).

**Table 1:** Primer sequences used in qPCR

Gene	Transcript_id		Primer Sequence (5′−3′)	Amplicon length
*LEF1*	ENSMUST00000106341	Forward	GGCATCCCTCATCCAGCTAT	99
		Reverse	TCTCTGTTCGTGTTGAGGCT	
*SMAD7*	ENSMUST00000174411	Forward	CGAGTTCATGCAGCAACCAT	122
		Reverse	TGAAGATGACCTCCAGCCAG	
* *LncRNA*	NONMMUT034790.2	Forward	AGTAACAACCCGGGAAGAGG	104
		Reverse	AGCGTTGGGAGTTTTGAACC	
*β‐Actin*		Forward	CGTTGACATCCGTAAAGACC	111
		Reverse	CTAGGAGCCAGAGCAGTAATC	

* http://www.noncode.org/show_rna.php?id=NONMMUT034790&version=2&utd=1#

### 2.10 Statistical analysis

All statistical analyses were performed using SPSS 16.0 software program (SPSS, Chicago, IL, USA). The qPCR data were analyzed using Student's *t* test to compare the means between the case and control samples. Pearson correlation was used in lncRNA–mRNA co‐expression analyses. Hypergeometric distribution test function was used to calculate the enrichment of functional terms in the annotation of co‐expressed mRNAs. A *p*‐value <0.05, fold enrichment >2, and log_2_FC >1 were considered statistically significant.

## 3 RESULTS

### 3.1 Morphology and histology of embryonic palate shelves

**Figure 1:**
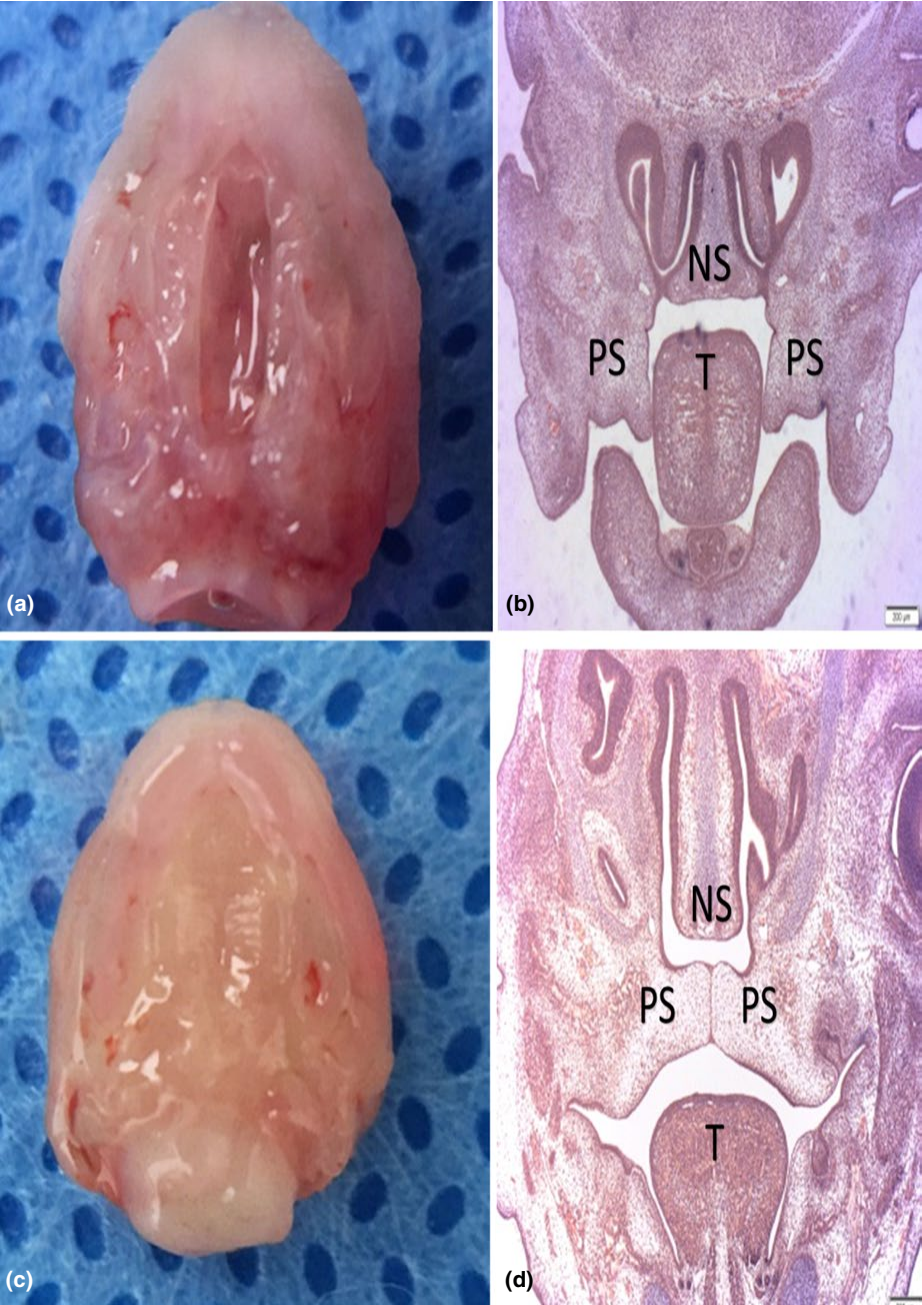
Morphology and histology (H&E) of palate shelves tissues at E14.5 between case versus control. (a,b) The palatal shelf separated without fusion of case. (c,d) The palatal shelf contacts the midline and has been fused. (a,c) Morphological specimens; (b,d), H&E staining results; PS, palatal shelf; SP, secondary palate; T, tongue; NS, nasal septum; H&E, hematoxylin and eosin

Embryonic palate shelf tissue was collected from pregnant mice. The palate shelf tissue and histological sections of the control group showed that the palatal shelf contacted the midline and had been fused through by formation of the midline epithelial seam (MES) in the midanterior region at E14.5, whereas those of the case group showed that the palatal shelf was separated without fusion (Figure 1).

### 3.2 Overview of mRNA and lncRNA expression profiles

**Figure 2:**
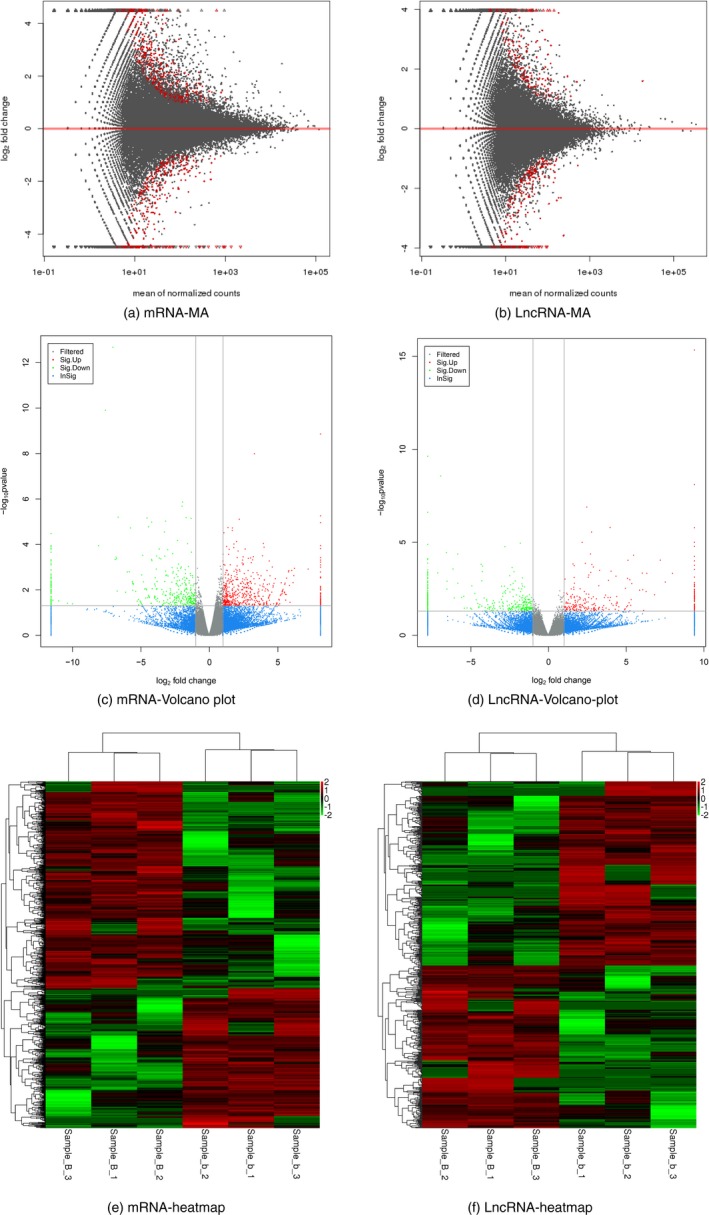
(a,b): The difference mRNA and lncRNA produced by the comparison is reflected in the MA plots. The x‐axis is the mean of normalized counts of all samples expression, and the y‐axis is log_2_FoldChange. The red plots are marked by a significant difference gene. (c,d): Differential expression analyses of mRNA and lncRNA between cases versus controls. The expression level for each gene was included in the volcano plot. Red and green points indicated the differential expression genes (DEGs). Gray and blue points indicated the non‐DEGs. Y‐axis contains the Log_10_ *p *value of the genes' mean expression level modified by DEseq package and x‐axis indicates Log_2 _of the fold changes among two libraries. (e,f): Result of hierarchical clustering for the differential genes, the red indicates high expression genes, and green expresses low expression genes (*p* < 0.05, log_2_FC >1)

A total of 703 differentially expressed mRNAs and 583 differentially expressed lncRNAs were identified (*p* < 0.05, log_2_FC >1). The differential expression of mRNAs and lncRNAs was compared using an MA plot (Figure 2a,b), and the expression level for each mRNA and lncRNA was represented using a volcano plot (Figure 2c,d). In order to reveal the correlation between mRNA and lncRNA expression profiles of CP‐related genes in the case and control groups, the differentially expressed mRNAs and lncRNAs were used to perform a heat map‐based unsupervised hierarchical clustering analysis (Figure 2e,f).

### 3.3 Correlation analysis of mRNA–lncRNA co‐expression

A total of 584 lncRNAs relative to mRNAs was identified (*p* < 0.05 and COR >0.7). Among the mRNA–lncRNA co‐expression, *NONMMUT034790.2 *relative to *SMAD7* had a *p* = 0.028, COR = 0.87, and *NONMMUT034790.2* relative to *LEF1* had a *p* = 0.037, COR = 0.84 (Supporting information Table S1).

### 3.4 The mRNA–lncRNA function annotation

**Figure 3:**
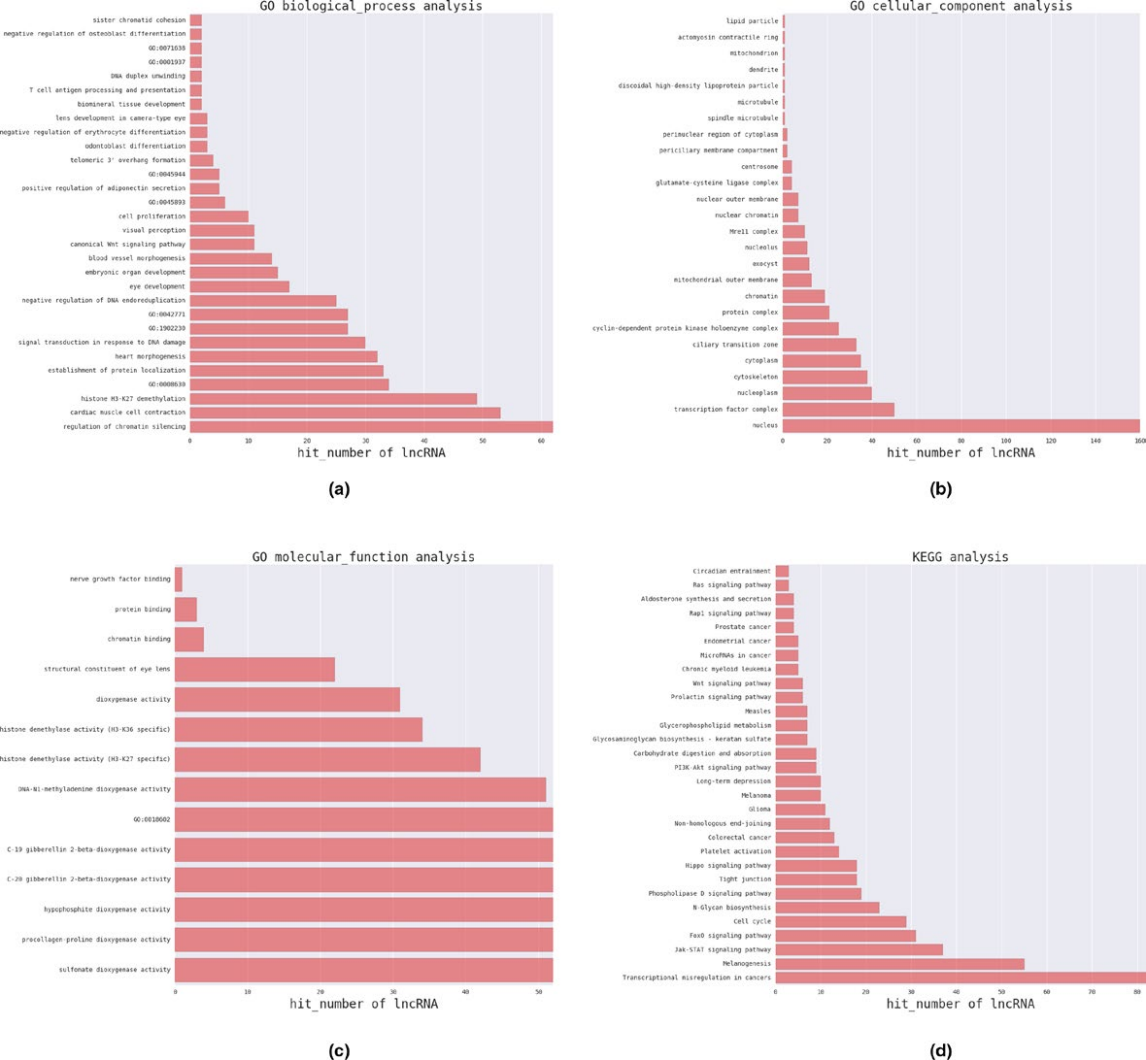
lncRNAs‐mRNAs co‐expression for function annotation. The GO and KEGG pathway enrichment analysis (500 counts) differentially expression lncRNA–mRNA co‐expression, including (a): biological processes; (b): cellular components; (c): molecular functions; (d): KEGG pathway

Pearson's correlation coefficient analysis was performed using the lncRNA–mRNA‐seq data, in which co‐expressed mRNAs of lncRNAs were identified (*p* < 0.05). The lncRNA function annotation was used in GO/KEGG pathway analyses by selecting the reliability prediction terms (*p* < 0.05, fold enrichment >2). A total of 500 counts of gene enrichment GO terms were obtained (Figure 3a, Supporting information Table S2)). The results of lncRNAs for GO analyses showed that lncRNA was associated with “transcriptional regulation,” “the canonical Wnt signaling pathway,” “positive regulation of transcription, DNA‐templated,” and “embryonic organ development.” KEGG pathway analysis indicated that the lncRNAs were involved in regulation of the “Hippo signaling pathway,” “*TGF‐β* signaling pathway,” “Wnt signaling pathway,” and “chromatin silencing” (Figure 3d, Supporting information Table S3). We then specifically analyzed and identified *NONMMUT034790.2* which is related to a biological process or molecular function. *NONMMUT034790.2* was involved in “negative regulation of transcription by competitive promoter binding” and “positive regulation of transcription, DNA‐templated” (*p* < 0.05, fold enrichment >2).

### 3.5 Constructed “lncRNA–TF‐target gene” *trans‐*regulatory network

**Figure 4:**
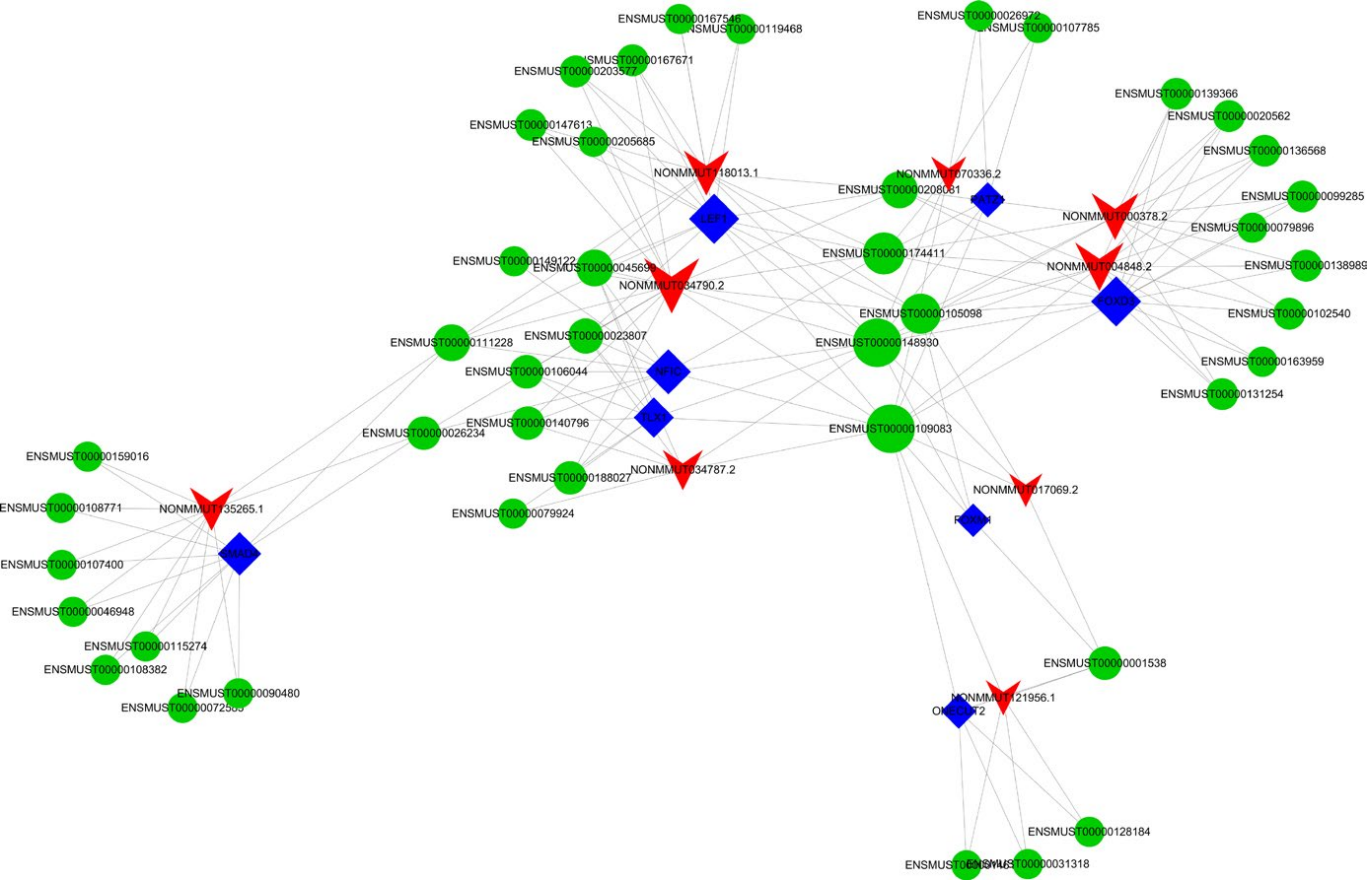
lncRNA–TF‐target gene *trans*‐regulatory network. The blue node represents the transcription factor, the red node represents the lncRNA, and the green node represents the target gene

It is universally known that *trans*‐regulatory mechanisms are involved in TF‐mediated chromatin regulation and transcription. We calculated the lncRNA–mRNA co‐expression profiles of chromatin regulators and TFs using the ENCODE database (https://www.encodeproject.org/) (Gerstein, et al., 2012; Guttman & Rinn, 2012; Khalil, et al., 2009) to identify common genes involved in lncRNA regulation. The “TF–lncRNA” regulatory network was generated using Cytoscape software (Supporting information Figure S1). Our results indicated that the *LEF1‐NONMMUT034790.2* was located at the center of the network map between the case and control groups (Supporting information Figure S1). According to the results of lncRNA–mRNA co‐expression analysis, we then constructed “lncRNA–TF‐target gene” *trans*‐regulatory network using Cytoscape software (Figure 4). The results indicated that the *NONMMUT034790.2‐LEF1‐SMAD7* was located at the center of the network map between case and control groups (*p* < 0.05, log_2_FC >1) (Supporting information Figure S1).

### 3.6 qPCR validation of TF, lncRNA, and target gene expression

**Figure 5:**
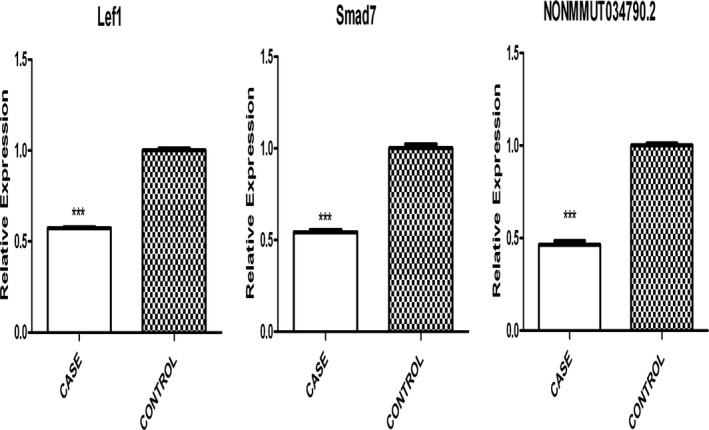
Relative levels of mRNA and lncRNA at mouse E14.5 in cases versus controls, as assessed using qPCR and then normalized to the housekeeping gene (*β*‐actin) (***p < 0.001)

qPCR was performed for further validation of selected differentially expressed lncRNA–mRNA to assess the correlations among “lncRNA–TF‐target gene” (*NONMMUT034790.2‐LEF1‐SMAD7*). We found that the expressions of *NONMMUT034790.2* (*p* = 4E‐07), *LEF1 *(*p* = 5E‐06), and *SMAD7 *(*p* = 6E‐05) mRNA were significantly down‐regulated in the case samples compared to that in the control samples (*p* < 0.05) (Figure 5). These data supported the lncRNA‐seq and mRNA‐seq data of the selected genes (Supporting information Tables S4, S5).

## 4 DISCUSSION

In the present study, we investigated whether aberrant lncRNAs have potential effects on embryonic mouse palate shelf fusion during palatal fusion in ATRA‐induced mouse CP. To verify this hypothesis, we established a CP model in C57BL/6 J mice after treatment with ATRA. Then, mRNA–lncRNAco‐expression profile analysis was performed at E14.5 palatal tissues to assess lncRNA regulatory mechanism during palatogenesis. Our results showed that the *NONMMUT034790.2‐LEF1*‐*SMAD7* co‐expression *trans*‐regulatory network might be associated with CP.

Altered gene expression and signaling in cells and tissues can be due to mutations and/or epigenetic regulation, such as aberrant lncRNAs. lncRNAs are functional regulators in various biological processes, including X‐chromosome inactivation (Panning and Jaenisch 1998; Sun, et al. 2006), cell differentiation, and the maintenance of cell identity (Sleutels, et al. 2002). lncRNAs are also known to regulate pluripotency during embryonic stem cell development by regulating chromatin structure and nuclear tissue (Deuve & Avner, 2011). lncRNAs are involved in the epigenetic regulation of transcription by mediating interactions between chromatin and proteins. lncRNA is composed of multiple binding modules, and thus, the epigenetic modifier or TFs can be combined to coordinate the recruitment into *cis* and *trans*‐specific genomic loci (Roberts, et al. 2014). In humans, CP is one of the major congenital defects with complex genetic and environmental etiology. However, few studies have demonstrated the regulation of lncRNA on palatal fusion, especially the ones that were involved in the *trans*‐regulatory mechanism. Our current study profiled mRNA–lncRNAco‐expression networks by Pearson's correlation coefficient analysis and identified a “lncRNA–TF‐target gene” network, which might directly regulate palatal fusion in ATRA‐induced mouse CP model. “lncRNA–TF‐target gene” and the implication of these aberrantly expressed lncRNA in cleft palate formation were assessed. Then we confirmed our data using qPCR.

In this study, the *trans*‐regulatory mechanism of *NONMMUT034790.2‐LEF1‐SMAD7* in palatogenesis following ATRA‐induced CP formation was demonstrated in three ways. (a) Changes in *NONMMUT034790.2‐LEF1‐SMAD7 *co‐expression were related to CP. (b) Function annotation analyses of GO/KEGG by Pearson's correlation coefficient analysis showed that *NONMMUT034790.2‐LEF1‐SMAD7 *is significantly enriched in important biological processes related to CP (Figure 3). (c) qPCR results showed that co‐expression level of *NONMMUT034790.2‐LEF1‐SMAD7* consistent with mRNA–lncRNA‐seq data.

The dysfunction of lncRNAs might contribute to CP. Therefore, the integrated analysis of the differentially expressed lncRNA and mRNA co‐expression could reveal the pathogenesis of CP. Among the different genes involved in EMT, *LEF1 *appears to be required for the induction of genes responsible for periderm transition into mesenchymal tissue and subsequent palate formation (Scapoli, et al. 2010). *LEF1* promotes palatal EMT, and *TGF‐β3* stimulates *LEF1* mRNA synthesis in MEE cells (Nawshad and Hay 2003; Nawshad, Medici, Liu, & Hay, 2007). These studies demonstrated that the activation of *LEF1* by *TGF‐β3* is a key step for the correct flow of events. *TGF‐β3* signaling has been shown to promote transcription of the *LEF1* gene in these cells through a *SMAD*‐dependent mechanism. Moreover, *SMAD7* interacts with *LEF1* through transcriptional regulators in Wnt signaling and induced apoptosis in a *TGF‐β*‐dependent manner (Edlund, et al. 2005). It also acts as an important positive regulator of *TGF‐β*‐induced EMT (Park, et al., 2015). Wang et al. showed that overexpression of *SMAD7* could enhance the EMT weakened by miR‐424‐5p mimics (Feng, Wang, Yang, Chen, & Wang, 2016). *TGF‐β3*, a member of the *TGF‐β* superfamily, is an essential growth factor that promotes palatogenesis (Jin, et al., 2015; Taya, O'Kane, & Ferguson, 1999). *TGF‐β* is a classical inducer of EMT. The expression of *TGF‐β* mRNA and protein shows restricted spatial–temporal patterns during palatal growth and remodeling (Degitz, Morris, Foley, & Francis, 1998), and *TGF‐β3* mutation contributes to CP in mice (Proetzel, et al. [Link]).

To our knowledge, this is the first study of the *trans*‐regulatory mechanism of “lncRNA–TF‐target genes” for ATRA‐induced CP. In our current study, we identified reduced expression of *LEF1,* *NONMMUT034790.2,* and *SMAD7* in CP mice, supporting the notion that these genes could inhibit EMT of the MEE through *trans‐*regulatory mechanism of “lncRNA–TF‐target genes” resulting in CP during palatal fusion. However, our current study is preliminary and needs further research to completely understand the relationship of gene alterations in CP formation. Our sample size was relatively small, and the palatal shelves were obtained directly from embryonic mouse tissues, and target tissue contamination with surrounding tissue may have occurred. Although *LEF1‐NONMMUT034790.2‐SMAD7* during palatal fusion in ATRA‐induced mouse CP was identified in our study, the underlying mechanisms of how “lncRNA–TF‐target gene” affect palatal fusion remains to be identified. We expect to integrate lncRNAs into the *trans*‐regulatory network, which will help us to understand the transcriptional control of TFs.

## 5 CONCLUSIONS

In summary, our results revealed that “lncRNA–TF‐target gene” play a role in ATRA‐induced CP. The *trans*‐regulatory network of *LEF1‐NONMMUT034790.2‐SMAD7* will further enhance our understanding of lncRNA functions in CP and will have an impact on the development of novel epigenetic biomarkers for CP and new strategies for treating CP.

## 6 DATA AVAILABILITY

All the data generated or analyzed during this study are included in this published article.

## 7 AUTHOR′S CONTRIBUTION

S.Y.S: Conception and design. X.S: Writing and revision of manuscript. H.Q.C: acquisition, analysis and interpretation of data. All authors wrote, reviewed, and/or revised the manuscript.

## DISCLOSURE

The authors have declared that no competing interests exist.
