# Supplementary figures and images for: A novel lncRNA‐mediated trans‐regulatory mechanism in the development of cleft palate in mouse
Source: Mol Genet Genomic Med. 2018 Dec 12;7(2):e00522. doi: 10.1002/mgg3.522 (PMC6393661; doi:10.1002/mgg3.522)

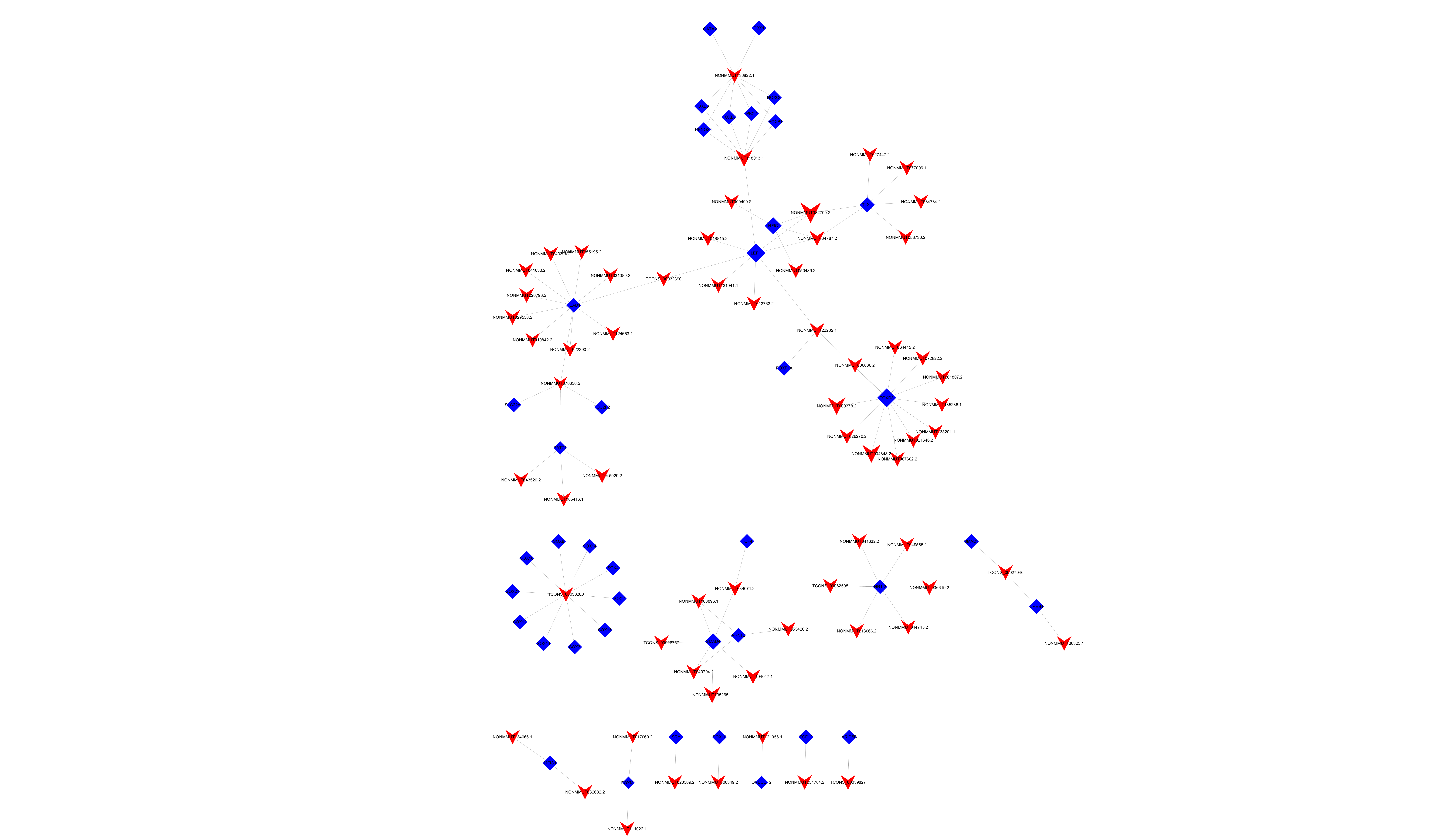

Supplement: Supplementary file 1 [file MGG3-7-na-s001.png]
